# Cerebellar hemorrhage in a healthy young adult: a case report

**DOI:** 10.1186/s13256-022-03584-4

**Published:** 2022-10-19

**Authors:** A. H. Ibrahim, N. Mohamad, T. A. Mohd Yusof Rasid, M. S. Abdullah

**Affiliations:** 1 Department of Radiology, School of Medical Sciences, Universiti Sains Malaysia, Jalan Raja Perempuan Zainab II, Kubang Kerian, 16150 Kota Bharu, Kelantan Malaysia; 2 Department of Diagnostic Imaging, Hospital Raja Perempuan Zainab II, 15586 Kota Bharu, Kelantan Malaysia; 3 Department of Radiology, Hospital Universiti Sains Malaysia, Kubang Kerian, Kota Bharu, Kelantan Malaysia

**Keywords:** Venous angioma, Cavernoma, Cerebellar hemorrhage, Familial cavernous hemangioma

## Abstract

**Background:**

Cavernous venous malformation is an uncommon entity that occurs in around 0.5% of the general population. Cerebellar cavernous venous malformation accounts for 1.2–11.8% of intracranial cavernous venous malformation cases. Patients are commonly asymptomatic until a hemorrhage occurs. In approximately 20% of the cases, cavernous venous malformation and developmental venous anomalies occur together, called mixed vascular malformation. Our case report reveals the imaging features of the mixed vascular malformation and highlights the appropriate imaging modality and sequence to detect the abnormalities.

**Case presentation:**

We report the case of a 15-year-old Malay male, a healthy young male who presented with dizziness, vomiting, and mild headache for 1 month. Computed tomography brain imaging at presentation revealed cerebellar hemorrhage with multiple cavernous venous malformation and coexisting developmental venous anomalies, which was then confirmed by magnetic resonance imaging. The patient was started on dexamethasone 4 mg four times a day, observed in the ward, and discharged well without neurological sequelae.

**Conclusion:**

A cavernous malformation with concurrent developmental venous anomalies requires accurate diagnosis. Our case report contributes to the literature on the imaging diagnosis of this disease, which is beneficial for current and future reference.

## Introduction

Cerebral venous malformations (CVM), is also known as cavernomas or cavernous hemangiomas, are not uncommon cerebral vascular malformations. According to Zyck and Gould [1], this vascular malformation is characterized by a group of abnormal and hyalinized capillaries without intervening brain tissue in between. These lesions are typically slow flow; hence, rupture risk is lower than with other venous malformations [1].

Most of the time, cavernoma is diagnosed incidentally. However, sometimes, patients may present with headaches, seizures, focal neurological deficits, or intracranial hemorrhages [1]. In a review article by Mouchtouris [2], the annual hemorrhage rate secondary to this lesion is 0.7–1.1%. The risk is higher, 4.5%, in patients with a history of bleeding [2]. The location of the lesion predicts the risk of rupture, with a higher risk in infratentorial and deep location of the lesion [1].

Multiple modalities are available to diagnose CVM. The advancement of noninvasive imaging technology has facilitated the diagnosis of vascular malformation [1]. Another slow-flow abnormality associated with CVM is developmental venous anomalies (DVAs), a congenital anomaly that occurs sporadically [3]. Here, we describe a case of multiple cerebellar CVMs with DVA in a young patient, focusing on the clinical presentation of the disease and diagnostic imaging characteristics.

## Case presentation

A 15-year-old Malay male presented with dizziness and vomiting for 1 month associated with a mild headache. He sought medical attention at general practitioners a few times for persistent symptoms. Otherwise, he denied limb weakness, imbalance gait, seizure, blurred vision, and tinnitus. He is a well-performing student with no history of substance use or previous trauma. On examination, the patient achieved the maximum Glasgow coma scale (GCS) score with no focal neurological deficit.

**Fig. 1:**
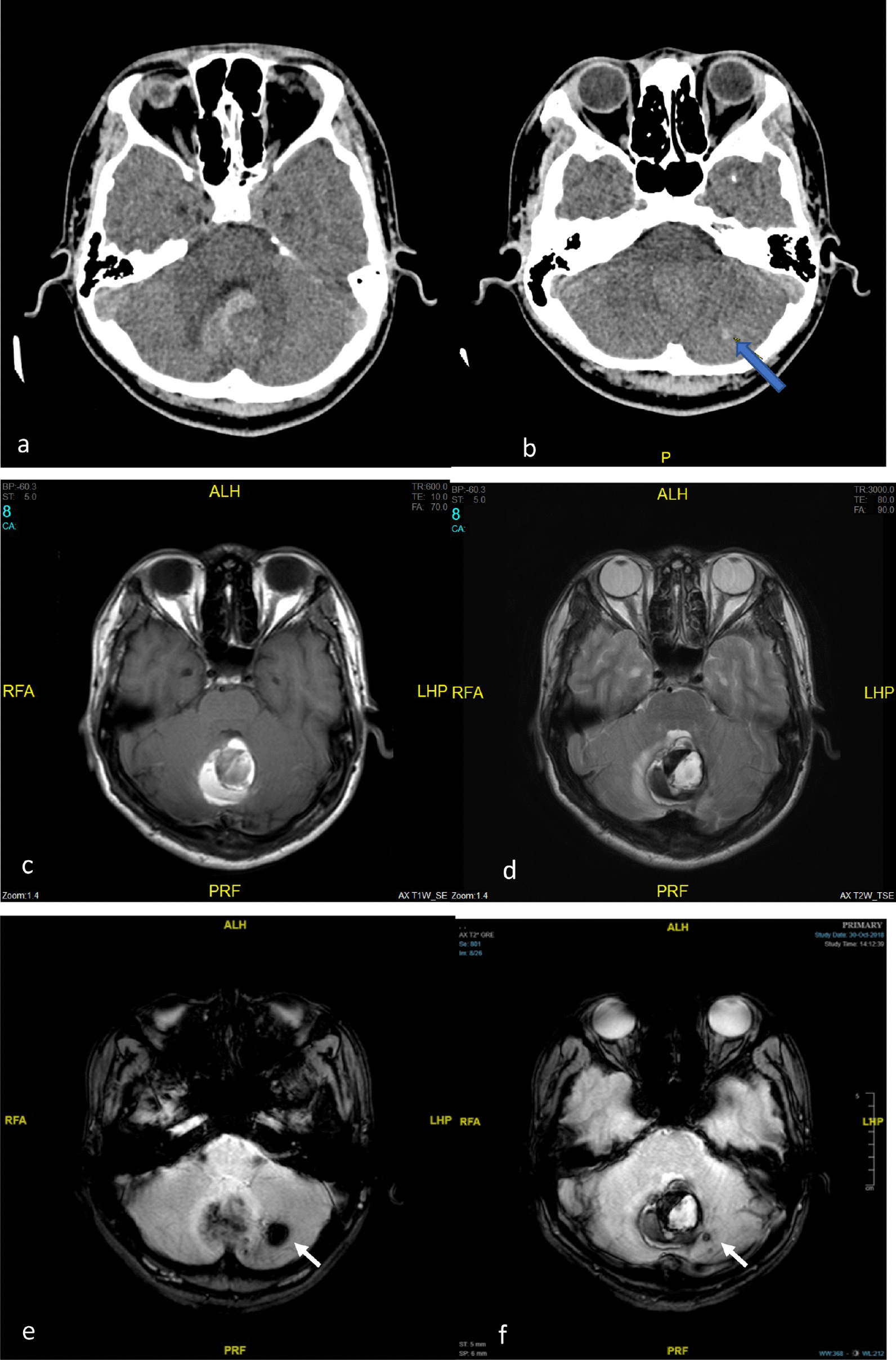
Non-contrast computed tomography brain (**a**) demonstrating cerebellar vermis hemorrhage. Contrasted computed tomography brain (**b**) showing enhancing area at left cerebellum, adjacent to the hemorrhage. Axial T1-weighted magnetic resonance imaging (MRI) (**c**), axial T2-weighted magnetic resonance imaging (**d**), and axial gradient echo sequence (**e**, **f**) demonstrating different ages of hemorrhage at cerebellar vermis. Multiple well-defined rounded blooming artifacts of varying sizes are seen in the left cerebellum, adjacent to the hemorrhage, representing cavernomas (white arrow in **e** and **f**)

This patient presented to a district hospital in October 2018 with a terrible headache. Urgent computed tomography (CT) of the brain showed cerebellar hemorrhage with perilesional edema (Fig. 1). The bleeding was located at the cerebellar vermis and caused a mass effect on the fourth ventricle and bilateral cerebellar peduncle. In addition, there were multiple small enhancing lesions in the cerebellar region adjacent to the hemorrhage (Fig. 1).

**Fig. 2:**
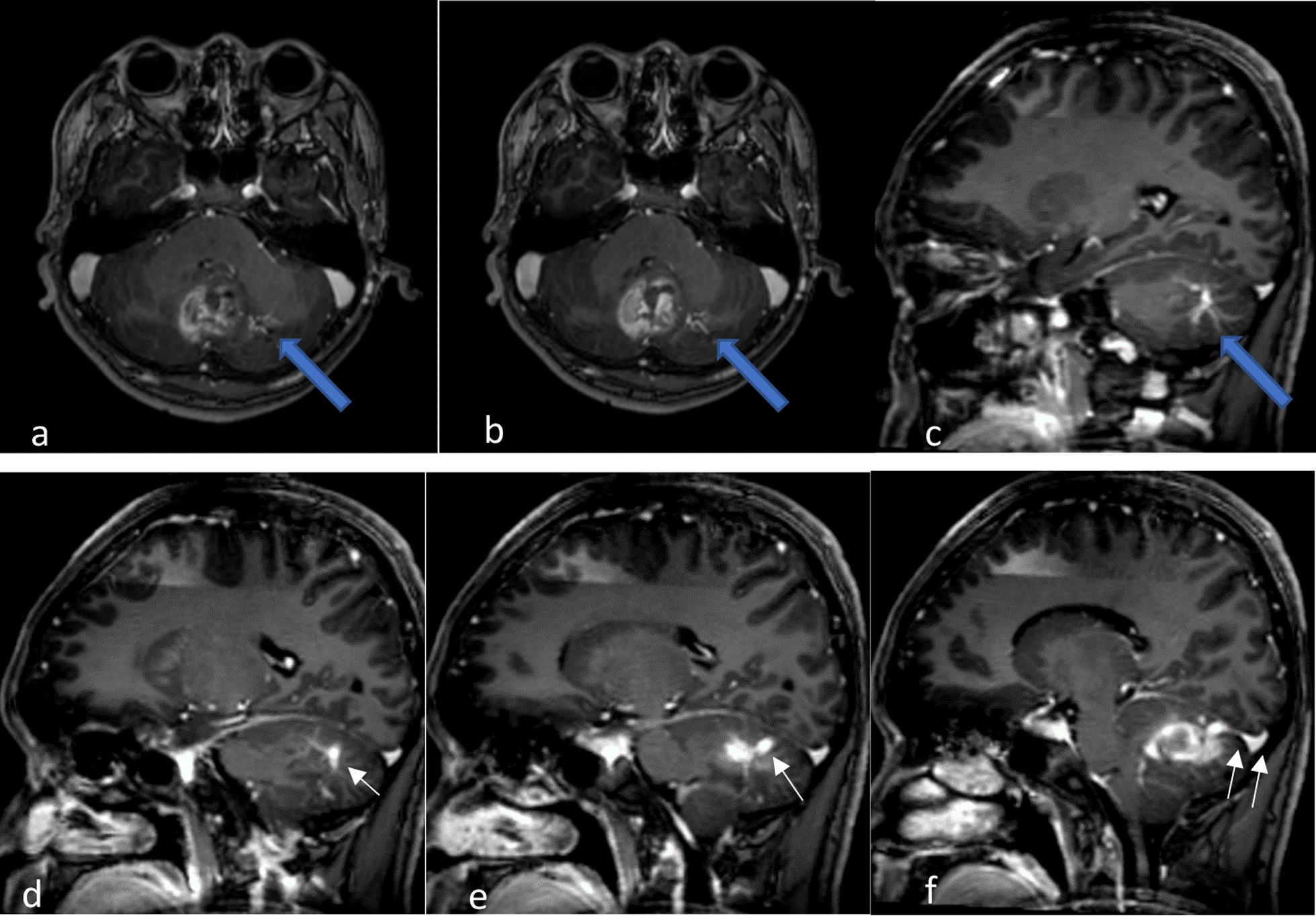
Axial post-contrast MRI (**a**, **b**) and sagittal MRI (**c**) showing enhancing tubular structure at left cerebellum resembling caput medusa sign (blue arrow in **a**–**c**) in keeping with developmental venous anomalies (DVAs). Note that the veins drain centrally toward a single draining vein (white arrow in **d**–**f**), draining into the torcular Herophili

He was immediately referred to the neurosurgical unit of Universiti Sains Malaysia (USM) Hospital and admitted to this unit for further management. Magnetic resonance imaging (MRI) of the brain performed 4 days later showed features of acute hemorrhage (Fig. 1). Numerous well-defined, rounded blooming artifacts of varying sizes were seen in the left cerebellum, adjacent to the hemorrhage, favoring cavernomas. A tubular tangle of blood vessels was seen giving rise to the “caput medusae” sign (Fig. 2). A large vein was seen from this region draining into the straight sinus. The patient was started on dexamethasone 4 mg, four times a day (QID), observed in the ward, and discharged well afterwards. On follow-up, the symptoms were resolved. The patient was offered surgical intervention, but he refused. He was well under neurosurgical follow-up until he relapsed 10 months later.

## Discussion

Cavernous venous malformation (CVM) occurs in different age groups, ranging from pediatric to elderly, with no gender predominance [4]. CVM occurs in supratentorial and infratentorial regions, more often seen in the supratentorial area [5]. Multiple lesions are found in up to 18.7% of cases and tend to be familial cavernomas [5]. Meanwhile, solitary CVM tends to be sporadic.

The typical presentation of CVM is seizure, progressive neurological deficits, hemorrhage, and headache. As many as 21% of the patients are asymptomatic. Seizure is the most common clinical presentation in most studies, while hemorrhage occurs in 10–23%.

Imaging findings in CVM depend on the blood degradation product present in the lesion. CT is essential for initial imaging to identify intracranial hemorrhage. However, CT findings for cavernoma are nonspecific. Multiple studies have shown low specificity of CT scan for CVM diagnosis [6]. Meanwhile, MRI is superior for lesion characterization. Digital subtraction angiography (DSA) of cerebral vessels also has a role in diagnosing associated lesions with cavernoma, such as developmental venous anomaly, bleeding tumor, or capillary telangiectasia.

CT brain plain typically demonstrates hyperdense and, less commonly, mixed hyperdense and hypodense lesions. The additional findings include intralesional calcification, poor contrast enhancement, and minimal mass effect. A round-shaped, well-defined margin and slightly uneven high density and absence of surrounding edema may also be features that indicate cavernoma. However, these findings are nonspecific, leading to difficulty in differentiating cavernoma and other lesions such as partially calcified avascular low-grade glioma [6].

MRI is a modality of choice to diagnose cavernous malformation. This malformation demonstrates a characteristic “popcorn” or “mulberry” appearance with a rim of signal loss due to hemosiderin or ferritin rim. Typical CVM is a mixed-signal lesion because it contains blood products from different ages. Usually, CVM does not demonstrate mass effect except for rapid growth or intramural hemorrhage. This is due to the nature of this lesion, which is a type of benign vascular hamartoma. MRI T2-weighted images (T2WI) are sensitive and specific in diagnosing cavernous malformation [7].

On T1-weighted images (T1WI), MRI demonstrates variable signal intensity based on the age of blood products. This reflects the slow flow, stagnation, or thrombosis of varying stages. In addition, a low fluid–fluid level may be evident. On gradient-echo (GRE) sequence, blooming artifacts are more apparent. This sequence is beneficial in detecting small lesions missed by the conventional spin-echo (SE) sequence [8]. Labauge *et al*., in 1998, suggested a gradient-echo sequence to diagnose cavernoma owing to its high sensitivity, demonstrating more significant signal loss in CVMs, particularly on GRE sequence, compared with conventional SE sequence [9]. Subsequent studies from Brunereau *et al*. [10] and Lehnhardt *et al*. [11] support this finding. No enhancement was observed in contrast study or CT study [8].

A cavernous malformation is a slow-flow lesion, rendering it challenging to detect using conventional digital subtraction angiography (DSA). CVM has therefore classically been defined as angiographically occult vascular malformations. However, in conjunction with flat-panel technology, recent technology has allowed the combination of cone-beam CT (CBCT) and DSA. This combination of technology has produced a high-resolution CT-like reconstruction of datasets obtained by conventional DSA. In a case report produced by Radvany in 2015, this recent technology documented a CVM and its associated DVA in three patients [12].

Mixed vascular malformations is a terminology used to describe concurrent CVMs and DVAs. This condition is observed in 20% of cases (range 2–40%) [13]. CT and MRI demonstrate numerous vascular structures radiating from the cerebellar lesion in this patient, suggestive of DVAs associated with cavernoma. DVAs are congenital, slow-flow venous anomalies. It consists of many dilated veins that converge into a single vein, giving a classic caput medusae or palm-tree appearance. This vein is usually seen merging into the dural sinus or ependymal vein.

In a study by Hon *et al*. [13], most patients (61%) were diagnosed with DVA incidentally. A non-hemorrhagic focal neurological deficit occurs in 6% of people, and symptomatic hemorrhage in 6%. The other presentation includes epileptic seizures and associated infarction [13].

Symptomatic CVMs with hemorrhage, edema, mass effect, and epilepsy may warrant surgery. Surgical management is currently indicated in cases of conspicuous bleeding for patients with neurological symptoms and intractable epilepsy. Surgeons need to know the presence of associated DVAs, since incorrect cauterization of the collecting vein may cause venous infarction [14].

## Conclusion

A cavernous malformation with concurrent DVA is an uncommon condition requiring accurate diagnosis. The management depends on number and location of cavernoma and hemorrhagic cases. The treating surgeon must be aware of the associated CVM and DVA as management may differ.

## Data Availability

All data analyzed during this study are included in this published article.

## Abbreviations

CVM: Cavernous venous malformation
CT: Computed tomography
DVA: Developmental venous anomalies
MRI: Magnetic resonance imaging
GCS: Glasgow coma scale
QID: Quarter in die; four times a day
DSA: Digital subtraction angiography
T2WI: T2-weighted images
T1WI: T1-weighted images
GRE: Gradient-echo MRI sequence
